# The impact of corruption on resident entrepreneurship behavior and its mediating effect analysis: An empirical study based on China

**DOI:** 10.1371/journal.pone.0317814

**Published:** 2025-04-04

**Authors:** Yifeng Zhu

**Affiliations:** School of Economics and Management, Jiangsu Vocational College of Finance and Economics, Huai’an, P.R. China; University of Professional Studies, GHANA

## Abstract

This study utilizes data from the 2015 China General Social Survey (CGSS) to investigate the impact of public sector corruption on resident entrepreneurship, including an analysis of heterogeneity among different types of entrepreneurs and the mediating effects of social networks and social trust. The findings reveal that while corruption may exhibit a certain inflection point effect on entrepreneurship, this effect is statistically insignificant due to the relatively low threshold of corruption. Overall, increasing levels of corruption significantly inhibit entrepreneurial choices among residents, particularly affecting “survival-oriented” and “out of system” entrepreneurs compared to “opportunity-oriented” and “within system” entrepreneurs. Furthermore, the study identifies that both objective social networks and subjective social trust serve as significant partial mediators in the relationship between corruption and entrepreneurship. Based on these insights, the paper recommends implementing robust anti-corruption measures, enhancing support for survival-oriented entrepreneurs, and fostering social trust to create a conducive environment for entrepreneurship.

## 1. Introduction

Entrepreneurship has served as a cornerstone for economic development globally, playing a particularly crucial role in China’s rapid economic transformation over the past 40 years. In recent years, however, China’s economic growth rate has decelerated, contrasting sharply with the high-speed growth experienced in the initial decades following the reform and opening up [1]. In response, former Premier Li Keqiang proposed the “Mass Entrepreneurship and Innovation” initiative to stimulate economic dynamism. In parallel, academic interest has intensified around the factors influencing entrepreneurial behavior in China. While significant strides have been made in identifying drivers of entrepreneurship, limited attention has been given to the impact of public officials’ corrupt practices on entrepreneurial activity, especially in a Chinese context. Although some studies do touch upon this subject, the literature on the mechanisms by which corruption influences entrepreneurship remains relatively underdeveloped [2–4].

Corruption influences entrepreneurship in complex ways, both obstructing and, under certain conditions, facilitating it. On one hand, corruption can raise entry barriers, increase transaction costs, and foster uncertainty, all of which discourage entrepreneurial activities, particularly among those lacking resources to navigate informal channels [5,6]. On the other hand, in certain contexts, corruption has been described as “greasing the wheels” by offering ways to circumvent bureaucratic inefficiencies [7,8]. In China, these mixed effects are especially pertinent given the nuanced relationship between entrepreneurs and public officials. However, little attention has been paid to how these dynamics vary across different types of entrepreneurs, such as “opportunity-oriented” versus “survival-oriented” groups, or those operating “within the system” compared to those working outside it.

This study addresses these gaps by examining the impact of corruption on resident entrepreneurship through three key contributions. First, unlike prior studies that tend to explore corruption’s impact at a broader macro level, this paper employs data from the 2015 China General Social Survey (CGSS) to assess how corruption affects entrepreneurial behavior on an individual level. This micro-level focus allows for a more nuanced understanding of how corruption impacts diverse groups. Second, this study incorporates both objective measures of corruption and subjective perceptions of social trust to capture the varied ways in which corruption can influence entrepreneurial decisions. Third, the study investigates mediating mechanisms, specifically social networks and social trust, to analyze how corruption indirectly shapes entrepreneurial choices. The mediation analysis is essential for understanding the pathways through which corruption exerts its influence, revealing that both the objective level of social networks and the subjective level of social trust have significant partial mediating effects.

The remainder of the paper is structured as follows: Section 2 reviews the literature on corruption and entrepreneurship, along with the hypotheses derived from this literature. Section 3 introduces the data and variables used for empirical testing. Section 4 outlines the econometric methodology, including the Probit model used to test the impact of corruption on entrepreneurial behavior. Section 5 delves into the heterogeneity analysis, examining how corruption’s impact varies across different entrepreneurial groups, as well as the mediating effect analysis. Finally, Section 6 summarizes the findings, presents policy recommendations and discusses limitations, emphasizing the need for stringent anti-corruption measures, improved regulatory mechanisms, and enhanced public trust.

## 2. Literature review and hypotheses

Recent advances in the theory of new institutional economics highlight the profound role that institutions play in shaping entrepreneurial activities and decision-making processes [9,10]. According to this theory, institutions—defined as the formal and informal rules governing behavior within a society—create the framework within which economic activities occur, significantly influencing both the incentives and constraints faced by entrepreneurs [11–13]. This theoretical framework underscores that well-functioning institutions reduce transaction costs, protect property rights, and promote trust, all of which are essential for entrepreneurial ventures. Conversely, when institutions are weak or compromised by corruption, they can distort market mechanisms and inhibit entrepreneurial activity [14]. Shleifer & Vishny (1993) argue that corruption often arises in environments where government officials possess significant discretionary power, enabling them to redistribute resources for personal gain. This power asymmetry allows officials to manipulate institutional rules, making corruption a tool that hinders fair competition and deters investment in entrepreneurship [15]. Rodriguez et al. (2006) expand on this by defining corruption as the abuse of public power to achieve personal benefits, noting that such behavior not only diverts resources but also imposes additional costs on businesses [16]. Muir & Gupta (2018) further emphasize that corruption fundamentally threatens the legitimacy of both state and market institutions, eroding trust and disrupting the regulatory environment that supports entrepreneurship. Therefore, from the perspective of new institutional economics, corruption can be seen as a barrier that weakens institutional frameworks, increases uncertainty, and discourages entrepreneurial innovation and risk-taking [17].

Most existing studies on public-sector corruption focus on its relationship with economic development, although no consensus has been reached. Mainstream economists often adopt the “harmful corruption theory,” asserting that corruption impedes economic growth, reduces investment, expands government size, and imposes significant social welfare costs [18–22]. However, some scholars argue that corruption can have positive effects on economic growth, giving rise to the “efficient corruption theory.” For example, in developing countries with complex regulatory environments, corruption can act as a lubricant for economic activities by enabling firms to bypass bureaucratic hurdles and expedite processes [7,23–25].

Despite substantial literature on the impacts of corruption, relatively few studies explore its effects on entrepreneurship, particularly on individual-level entrepreneurship. Some researchers suggest that corruption may facilitate entrepreneurial activity by easing credit constraints, enabling firms to secure policy support, and helping them avoid inefficient regulations [26–30]. For instance, empirical studies by Liu & Wang (2014) find that rent-seeking behaviors significantly increase firms’ R&D investments [31]. Similarly, Xu & Li (2016) show that corruption may stimulate private enterprises’ investment expenditures. However, the prevailing view holds that corruption negatively impacts entrepreneurship by hindering access to financing and elevating transaction costs [32]. For example, Nan (2009) illustrates that corruption reduces entrepreneurial efficiency by increasing financing costs [33], while Wang & Gao (2017) find that anti-corruption measures promote innovation in both state-owned and private enterprises. Other studies highlight the moderating role of external factors, such as legal system maturity and market economy level [34]. He & Chen (2018) show that the adverse effects of corruption on entrepreneurship are especially pronounced in regions with low financial development [35], while Liu (2023) finds that bank credit corruption raises capital costs, discouraging innovation investments [22]. Zhou et al. (2023) also report that anti-corruption policies alleviate corporate financing constraints and that corporate ownership structure and political connections play a moderating role [36]. Based on these insights, we propose the following hypothesis:

**Hypothesis 1:** Corruption has a non-linear impact on resident entrepreneurship, initially exerting a positive “lubricating” effect but eventually reaching a threshold where it begins to inhibit entrepreneurship.

The Global Entrepreneurship Monitor (GEM) survey introduced the concepts of “survival-oriented” and “opportunity-oriented” entrepreneurship in 2001. “Survival-oriented” entrepreneurship occurs when individuals pursue entrepreneurial activities due to limited employment options, while “opportunity-oriented” entrepreneurship involves individuals exploiting specific business opportunities [3,37,38]. In addition, China’s market-oriented reforms have fostered distinct economic structures: “within the system” and “outside the system.” Zhang et al. (2017) find that entrepreneurs from “within the system” backgrounds benefit from unique privileges regarding resource access [39]. Consequently, the impact of corruption on entrepreneurship likely varies across different entrepreneurial types and demographic groups. Based on this analysis, we propose:

**Hypothesis 2:** The effect of corruption on resident entrepreneurship varies significantly by entrepreneurial type, with differences observed between survival-oriented and opportunity-oriented entrepreneurs, as well as between those within and outside of the institutional system.

Greve & Salaff (2003), Yueh (2009), and Kerr & Mandorff (2023) demonstrate that social networks play a crucial role in entrepreneurship by providing access to financial capital, technical expertise, information, and emotional support [40–42]. Furthermore, corruption may erode societal trust, which is essential for fostering entrepreneurial activity. Zhou et al. (2015) suggest that trust facilitates entrepreneurship by reducing risk, promoting information exchange, and enhancing collaboration [43]. Therefore, the study explores the mediating role of social networks and social trust in the relationship between corruption and entrepreneurship, proposing:

**Hypothesis 3:** Social networks and social trust serve as critical mediators in the relationship between corruption and entrepreneurial behavior, with both objective network ties and subjective trust levels influencing the degree to which corruption impacts resident entrepreneurship.

## 3. Data and variable

### 3.1. Data source

The household microdata used in this study is drawn from the 2015 China General Social Survey (CGSS2015). CGSS2015 provides a representative sample of 28 provinces, municipalities, and autonomous regions (excluding Xizang, Xinjiang, Hainan, Hong Kong, Macao, and Taiwan), yielding a total of 10,968 valid responses. After excluding cases with incomplete data on relevant variables, this study analyzes a final sample size of 5,631.

Data on corruption are derived from the annual work reports of provincial and municipal procuratorates and the China Statistical Yearbook. Additional macroeconomic data are obtained from annual reports published by the National Bureau of Statistics. The macroeconomic and microeconomic data were matched and consolidated at the provincial level using CGSS2015 data.

### 3.2. Variable selection

#### 3.2.1. Dependent variable.

The dependent variable, household entrepreneurial behavior, is assessed based on responses to the CGSS2015 question, “Which of the following situations best describes your current employment status?” Respondents who selected “sole proprietorship” or “being a boss (or partner)” were classified as entrepreneurial households. The study further refines this classification by excluding cases where the reported number of employees is clearly unreasonable. The final dataset includes 790 entrepreneurial households, representing 14.03% of the sample.

#### 3.2.2. Core variable.

For the core variable, corruption, the study adopts the approach of Chen & Li (2012) [44], with modifications to account for a potential lagged effect and concerns of potential endogeneity. Specifically, the study measures corruption as the natural logarithm of the number of cases of corruption and dereliction of duty recorded in 2013, divided by the total number of public officials in the same year. This captures illegal activities by public officials in each province. Additionally, to explore potential non-linear relationships, the study includes the square term of the corruption measure.

#### 3.2.3. Control variables.

Following established research practices [43,45,46], the study incorporates a range of control variables, categorized as follows: (1) Household Demographic Characteristics: These include the age (Based on China’s labor policies and retirement age standards, the age ranges differs for males and females.), gender, and labor force participation rate of the household head. Given the potential for an inverted U-shaped relationship with age, the study includes both the age and the square of the age of the household head. (2) Household Sociological Characteristics: Variables in this category include marital status, health status, education level, household registration status, Communist Party membership, and religious beliefs of the household head. (3) Household Economic Characteristics: This set of variables includes household participation in social insurance, risk preference of the household head, household income for the previous year, the number of properties owned, and vehicle ownership status. (4) Macroeconomic Characteristics: These are measured at the provincial level and include per capita GDP, unemployment rate, and urbanization rate. (5) Regional Characteristics: To capture regional differences, the study uses the western region as the control group and introduces dummy variables for eastern and central regions. Definitions and descriptive statistics for these variables are provided in Table 1.

**Table 1:** Definition of main variables and descriptive statistics.

Variable	Definition	Obs	Mean	Std	Min	Max
entre	Indicator of entrepreneurial activity; 1 if the family is engaged in entrepreneurship, 0 otherwise	5631	0.14	0.35	0	1
corruption	Logarithm of the number of officials involved in corruption-related crimes per 10,000 public officials	5631	3.44	0.40	2.24	4.23
age	Age of the household head (male: 18-60 years, female: 18-55 years)	5631	41.08	10.96	18	60
age2	Square of the household head’s age	5631	1807.50	873.59	324	3600
gender	Gender of the household head; 1 for male, 0 otherwise	5631	0.51	0.50	0	1
marr	Marital status; 1 for married, 0 otherwise	5631	0.81	0.39	0	1
educ	Years of education completed by the household head	5631	9.83	3.97	0	19
health	Self-rated health status of the household head; scale from 1 (lowest) to 5 (highest)	5631	3.86	1.00	1	5
religion	Religious affiliation; 1 if the household head has religious beliefs, 0 otherwise	5631	0.10	0.31	0	1
commy	Communist Party membership; 1 if the household head is a member, 0 otherwise	5631	0.08	0.28	0	1
citz	Household registration type; 1 for non-agricultural, 0 otherwise	5631	0.42	0.49	0	1
insu	Participation in social security; 1 if the household head is insured, 0 otherwise	5631	0.64	0.48	0	1
risk	Risk preference of the household head; scale from 1 (lowest) to 6 (highest)	5631	1.12	0.41	1	6
labrate	Ratio of household labor force to total household population	5631	0.76	0.24	0.11	1
lfinc	Last year’s household annual income (logarithmic)	5631	10.58	1.56	0	16.10
house	Number of properties owned by the household; capped at 5 for five or more properties	5631	1.10	0.57	0	5
car	Car ownership; 1 if the household owns a car, 0 otherwise	5631	0.21	0.41	0	1
lmgdp	Logarithm of regional (provincial) per capita GDP	5631	1.64	0.39	0.96	2.37
unra	Regional (provincial) unemployment rate	5631	3.30	0.69	1.4	4.5
cira	Regional (provincial) urbanization rate (proportion of urban population)	5631	0.59	0.12	0.42	0.88
Area	Regional dummy variable; 1 for eastern region, 2 for central, and 3 for western	5631	1.92	0.75	1	3

## 4. Empirical analysis

### 4.1. Model

To account for the binary nature of the dependent variable, which represents household entrepreneurial choice, the Probit model is used in this study, as it is better suited than ordinary least squares (OLS) for binary outcome variables. This approach avoids the potential bias in estimation results that would arise from using OLS directly [47]. Following the methodology of prior research on entrepreneurial choice [46,48], the Probit model estimates the probability of a household engaging in entrepreneurship based on the independent and control variables discussed previously. The econometric model is specified as follows:


$$\text{Prob}\left({\text{entre}}_{\text{ij}}=1\right)=\text{F}\left({\text{β}}_{0}+{\text{β}}_{1}\times {\text{corruption}}_{\text{j}}+{\text{β}}_{2}\times {\text{corruption}}_{\text{j}}^{2}+{\text{β}}_{3}\times {\text{X}}_{\text{ij}}\right)$$ (1)

Among them, ${\text{entre}}_{\text{ij}}$ represents the binary variable of entrepreneurship for i households in the j-th province, where 1 represents entrepreneurship and 0 represents no entrepreneurship; ${\text{corruption}}_{\text{j}}$ represents the corruption and dereliction of duty rate of public officials in the j-th province (logarithmic), with a higher value indicating a higher degree of corruption; ${\text{X}}_{\text{ij}}$ represents the individual level, family level, and regional control variables that affect the entrepreneurial choices of i-households in the j-th province. In addition, in order to make the estimation results unbiased and effective, this paper adopts robust standard error for estimation.

**Fig 1:**
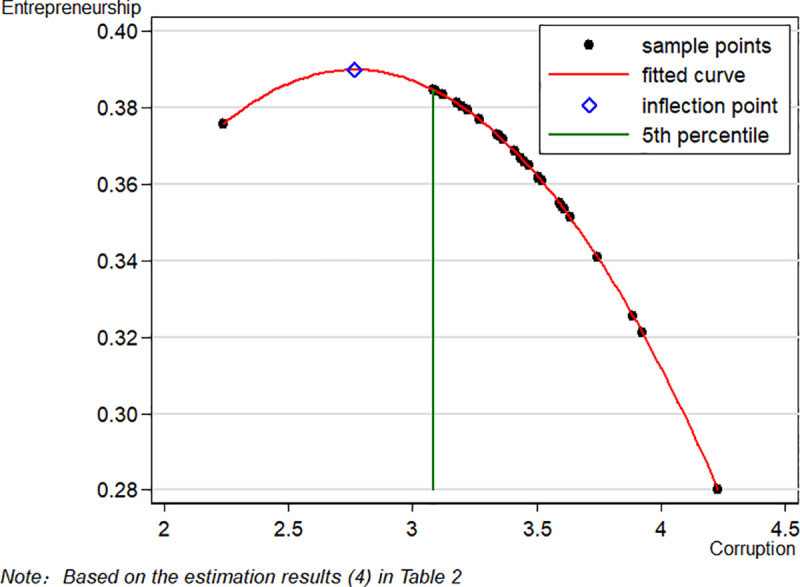
Quadratic relationship between corruption and entrepreneurship.

The regression results in Table 2 reveal the average marginal effects of each variable, along with their robust standard errors. Fig 1 depicts the quadratic relationship between corruption and entrepreneurship, showing both inhibiting and enabling impacts depending on the level of corruption. The findings suggest an inflection point effect between the rate of corruption and household entrepreneurship. Specifically, when corruption levels are low, there appears to be a minor positive effect on entrepreneurship. This aligns with the “grease the wheels” hypothesis [7,25], which argues that minor corruption can facilitate bureaucratic processes and enable entrepreneurs to expedite tasks such as license applications and access to certain privileges, ultimately accelerating entrepreneurial activity.

**Table 2:** Estimation results.

Entre	(1)	(2)	(3)	(4)
corruption	0.266^**^ (0.107)	0.259^**^ (0.102)	0.280^***^ (0.100)	0.282^**^ (0.126)
corruption^2^	-0.045^***^ (0.016)	-0.044^***^ (0.016)	-0.047^***^ (0.015)	-0.051^***^ (0.019)
age		0.030^***^ (0.004)	0.031^***^ (0.004)	0.029^***^ (0.004)
Age2		-0.000^***^ (0.000)	-0.000^***^ (0.000)	-0.000^***^ (0.000)
gender		0.024^**^ (0.009)	0.030^***^ (0.009)	0.032^***^ (0.009)
marr		0.108^***^ (0.016)	0.120^***^ (0.017)	0.089^***^ (0.017)
educr		0.000 (0.001)	-0.000 (0.001)	-0.003^**^ (0.001)
health			0.020^***^ (0.005)	0.011^**^ (0.005)
religion			0.048^***^ (0.013)	0.053^***^ (0.013)
Commy			-0.110^***^ (0.020)	-0.123^***^ (0.020)
citz			0.027^**^ (0.011)	0.026^**^ (0.011)
labrate			0.037^*^ (0.020)	0.039^**^ (0.019)
insu			-0.037^***^ (0.009)	-0.042^***^ (0.009)
risk			0.027^**^ (0.011)	0.006 (0.011)
lfinc				0.018^***^ (0.006)
house				0.017^**^ (0.008)
car				0.099^***^ (0.010)
lmgdp				0.031 (0.036)
unra				-0.018^**^ (0.008)
unra				-0.341^***^ (0.107)
east				0.016 (0.021)
middle				0.046^***^ (0.013)
Observations	5631	5631	5631	5630
Pseudo R^2^	0.003	0.047	0.067	0.112

Note:

* ,

** , and

***  represent significance at the 10%, 5%, and 1% levels, respectively, with corresponding standard errors in parentheses.

However, as corruption surpasses this threshold, the negative impact on entrepreneurship becomes pronounced, consistent with the “sand the wheels” theory. When corruption intensifies, the costs imposed on entrepreneurs—due to demands for bribes, increased regulatory hurdles, and diminished trust in public institutions—outweigh any potential gains from engaging in rent-seeking behavior. The discouraging social environment can also deter potential entrepreneurs, contributing to a significant overall decline in entrepreneurial activity. This observation is supported by the findings of Wang & Gao (2017) and Liu (2023) [22,34], who also report that high levels of corruption hinder business development by increasing financial and operational burdens.

On further examination, as illustrated in Fig 1, the inflection points calculated in the four regression results fall below the 5th percentile of the corruption variable. This finding implies that the inflection point effect is not particularly significant. Therefore, although there is an observable quadratic correlation between corruption and entrepreneurship, the supposed inflection effect lacks statistical significance, thereby partially refuting Hypothesis 1. Overall, deepening corruption significantly inhibits entrepreneurial behavior.

### 4.2. Endogeneity

To address concerns of potential endogeneity, commonly encountered in entrepreneurship literature, this study briefly discusses the issue. Endogeneity often arises due to omitted variables or reverse causality [47]. However, this analysis minimizes these risks because the dependent variable is the 2015 household entrepreneurial level, while the key independent variable is the 2013 corruption rate. This temporal separation mitigates the possibility of reverse causality. Additionally, factors influencing entrepreneurship in 2015 would not have likely impacted the corruption rate two years earlier. Therefore, the endogeneity concerns are not expected to be significant in this analysis.

### 4.3. Robustness test

To ensure the reliability of the results, this study conducted several robustness checks. These include replacing the two-year lagged corruption rate with a one-year lag, changing core variable definitions, modifying regression models, redefining control variables, and addressing outliers.

Firstly, change the method of measuring the level of corruption among public officials. We conducted factor analysis on the relevant survey questions in CGSS2015 using survey data from six questions: “How do you evaluate the level of integrity of various party and government officials” (CGSS2015 includes five questions regarding local national officials, police, judges, prosecutors, and overall evaluation. Answers 1-5 correspond to generally very corrupt, mostly corrupt, almost half clean, mostly clean, and generally very clean.) and “Overall, do you think today’s society is fair and unfair?” (Answers 1-5 correspond to completely unfair, relatively unfair, not fair but not unfair, relatively fair, and completely fair.). Based on this, a subjective indicator of residents’ perception of corruption towards society and government officials is constructed, in order to measure the impact of corruption on residents’ entrepreneurship from multiple perspectives. (The KMO test statistic value is 0.781, and the significance level of Bartlett’s sphericity test is less than 0.001, indicating that factor analysis is feasible.)

Additionally, the model was re-estimated using the Logit model, as a comparison to the Probit model results. The control variable for “household income in the previous year” was substituted with “family economic status” for robustness. Lastly, the total household income variable underwent a 2% trimming to remove potential outliers, and the model was re-estimated accordingly.

The robustness test results, displayed in Table 3, align closely with the original estimates, affirming the significance of the core explanatory variable—corruption rate and its square term—at the 1% level. The coefficients of other key explanatory variables remain consistent with previous results (not all results are reported due to space constraints), which strengthens the reliability of the primary findings.

**Table 3:** Robustness test.

Entre	Advance by 1 period	Perception of corruption	Logit model	Replace control variables	Remove extreme values
corruption	0.281^*^ (0.155)	-0.029^***^ (0.011)	0.252^**^ (0.117)	0.285^**^ (0.126)	0.282^**^ (0.126)
corruption^2^	-0.049^**^ (0.023)	——	-0.047^***^ (0.018)	-0.052^***^ (0.019)	-0.051^***^ (0.019)
control variable	Yes	Yes	Yes	Yes	Yes
Observations	5630	1940	5630	5631	5630
Pseudo R^2^	0.110	0.124	0.114	0.112	0.112

Note: For simplicity, only the results of core variables are reported here. The other notes are the same as Table 2.

## 5. Heterogeneity and mediating effect analysis

### 5.1. Heterogeneity analysis

To further examine the impact of corruption on resident entrepreneurship, this article conducts a heterogeneity analysis focusing on both entrepreneurial types and the structural characteristics of entrepreneurial households.

#### 5.1.1. Classification of entrepreneurial types.

Drawing on previous literature [3,37,38], this article defines “survival-oriented entrepreneurship” as entrepreneurial households with 0 to 7 employees (including 7), and “opportunity-oriented entrepreneurship” as households with 8 or more employees engaged in entrepreneurial activities.

As shown in Table 4, corruption has an insignificant impact on opportunity-oriented entrepreneurship but a significant negative effect on survival-oriented entrepreneurship. Opportunity-oriented businesses are typically larger in scale, whereas smaller, survival-oriented ventures often forgo formal business registration to minimize costs. Given their smaller size, survival-oriented businesses are more susceptible to the adverse influence of public officials’ illegal and irregular activities. Conversely, opportunity-oriented enterprises tend to be better equipped to counteract such negative impacts. These businesses are often launched by individuals who have already secured their basic living needs and pursue entrepreneurship to access greater business opportunities and returns. This entrepreneurial group also tends to possess a certain level of social capital, allowing them to better navigate the additional costs introduced by corrupt behaviors of public officials. Thus, the adverse effects of corruption are most pronounced within the survival-oriented entrepreneurship segment.

**Table 4:** Heterogeneity analysis I.

Entre	Survival oriented		Opportunity oriented	
corruption	−0.055^***^ (0.015)	0.293^**^ (0.124)	−0.002 (0.002)	0.019 (0.018)
corruption^2^	——	−0.052^***^ (0.018)	——	−0.003 (0.003)
control variable	Yes	Yes	Yes	Yes
Observations	5543	5543	4927	4927
Pseudo R^2^	0.095	0.097	0.291	0.293

Notes are the same as Table 3.

#### 5.1.2. Classification of system.

The term “within the system” generally refers to entities or groups that exercise governmental authority or benefit directly from state-owned assets. This includes three main categories: (1) all levels of party and government organizations that exercise state power, (2) public institutions such as schools, hospitals, and research institutions, and (3) state-owned or collectively controlled enterprises. In contrast, “outside the system” encompasses individual businesses, private enterprises, and foreign-funded companies that operate independently of government power or state-owned assets.

The results in Table 5 show that corruption has a significant negative impact on entrepreneurship among households classified as “outside the system.” Conversely, in column 3, corruption appears to have a positive effect on entrepreneurship within “system” households, though this effect is not statistically significant. This article proposes two main reasons for these findings. First, “outside the system” households generally lack the social resources and institutional advantages that “within the system” households enjoy, which limits their capacity to withstand policy shifts or market instability. As a result, they are more vulnerable to the adverse effects of illegal and irregular actions by public officials. Second, “within the system” households often occupy positions closely related to public officials and may even benefit from corrupt practices. As corruption intensifies, these households may feel more incentivized to capitalize on their positions by encouraging family members to pursue entrepreneurial ventures that can leverage such connections for profit.

**Table 5:** Heterogeneity analysis II.

Entre	Outside the system		Within the system	
corruption	−0.086^***^ (0.018)	0.310^**^ (0.150)	0.015 (0.018)	0.189 (0.147)
corruption^2^	——	-0.059^**^ (0.022)	——	−0.027 (0.023)
control variable	Yes	Yes	Yes	Yes
Observations	4616	4616	947	947
Pseudo R^2^	0.130	0.132	0.206	0.209

Notes are the same as Table 3.

These heterogeneity test results lend support to Hypothesis 2, confirming that the impact of corruption on entrepreneurship varies significantly depending on whether the household is “within” or “outside” the system.

### 5.2. Mediating effect analysis

To further explore the mechanisms by which corruption influences resident entrepreneurship, Hypothesis 3 proposes that both objective social networks and subjective social trust serve as mediating factors in the impact of corruption on entrepreneurial choices. Therefore, this section investigates these mediation effects individually.

The most widely used method for testing mediating effects is the stepwise procedure by Baron and Kenny (1986) [49], which offers a straightforward and interpretable approach. This method first examines the influence of the independent variable X on the dependent variable Y, and if X impacts Y through the mediator M, M is identified as a mediator. The relationships among these variables are described by the following regression equations:


$$Y=cX+e_{1}$$ (2.1)

$$M=aX+e_{2}$$ (2.2)

$$Y=c^{\prime }X+bM+e_{3}$$ (2.3)

Here, the coefficient c of equation (2.1) represents the total effect of X on Y; a of equation (2.2) captures the effect of X on M; b of equation (2.3) reflects the effect of M on Y, controlling for X; and c’ is the direct effect of X on Y after accounting for M; e1 ~ e3 are regression residuals. The mediating effect is defined as the product of ab. Given the enhancements in mediation effect testing and related theoretical advancements, this study adopts the latest mediation testing framework proposed by Wen & Ye (2014) [50].

It should be noted, however, that traditional mediation models assume continuous independent, mediator, and dependent variables. In this study, the dependent variable is a binary categorical variable, necessitating some specificity when addressing the product testing of coefficients [51]. Two key points are clarified here:

(1) If the sequential testing results are all significant, the mediating effect can be directly inferred without coefficient product testing. As this study’s mediation effect results satisfy this condition, no further coefficient product tests are required. Nevertheless, this study applies a coefficient product test as a verification measure.
(2) To accommodate categorical variable mediation models, recent studies have suggested using marginal probability product statistics for product testing. Given the growing consensus on substituting the binary Probit model with the LPM model [52–55], the OLS regression is re-estimated, and the Bootstrap method is applied with 1000 iterations for coefficient product testing.

#### 5.2.1. Social networks.

Social networks are typically analyzed in terms of breadth and depth. While prior literature often focuses on breadth [41,42], depth here is represented by the frequency of social interactions. As the CGSS2015 does not contain data on network breadth, this study solely explores the mediating effect of social network depth (Social depth is mainly measured based on the answer in the CGSS2015 questionnaire, “Have you often engaged in the following activities and gatherings with friends in your free time in the past year?” It is divided into 1-5 levels, and the higher the number, the higher the level, and the higher the social frequency.).

The mediation analysis using the Bootstrap test results (In the Bootstrap product test, the coefficient of the mediating effect of social networks is -0.0031, with a p-value of 0.012; The coefficient of direct effect is -0.0185, with a p-value of 0.039, indicating a significant mediating effect.) reveals that social networks partially mediate the relationship between corruption and resident entrepreneurship. The results in Table 6 show that increased corruption correlates with higher social interaction frequency among entrepreneurs, aligning with theoretical expectations. However, social interaction frequency has a significant negative impact on entrepreneurship, suggesting that increased socializing inhibits entrepreneurial activities. This finding diverges from previous literature on the positive role of social networks in entrepreneurship. This study posits that this discrepancy is attributable to the unique measure of network depth, as opposed to the social network breadth typically examined in past studies. Corruption-driven socializing often involves obligatory exchanges with public officials, which may impose substantial additional costs on entrepreneurs, deplete their focus, and create physical and psychological burdens, thereby hindering entrepreneurship.

**Table 6:** Mediating effect analysis I.

Dependent variable	Entre	Social networks	Entre
corruption	0.282^**^ (0.126)	0.087^***^ (0.029)	0.283^**^ (0.128)
corruption^2^	−0.051^***^ (0.019)		−0.054^***^ (0.018)
social networks			−0.089^***^ (0.025)
control variable	Yes	Yes	Yes
Observations	5614	5596	5596
R^2^	——	0.102	——
Pseudo R^2^	0.110	——	0.110

Notes are the same as Table 3.

#### 5.2.2. Social trust.

The analysis of social trust (Social trust is mainly measured based on the response in the CGSS2015 questionnaire, which states “trust in general social interactions/interactions that do not directly involve monetary benefits, towards strangers”. It is divided into 1-5 levels, with the higher the number, the higher the level, and the higher the level of trust.) as a mediating factor follows the same methodological steps as above. According to the Bootstrap test results (In the Bootstrap product test, the coefficient of the mediating effect of social networks is -0.0056, with a p-value of 0.006; The coefficient of direct effect is -0.0153, with a p-value of 0.074, indicating a significant mediating effect.), the results in Table 7 confirm a significant mediating effect of social trust on the relationship between corruption and entrepreneurship.

**Table 7:** Mediating effect analysis II.

Dependent variable	Entre	Social trust	Entre
corruption	0.283^**^ (0.126)	−0.165^***^ (0.022)	0.283^**^ (0.128)
corruption^2^	−0.052^***^ (0.018)		−0.053^***^ (0.019)
social trust			0.115^***^ (0.015)
control variable	Yes	Yes	Yes
Observations	5631	5546	5546
R^2^	——	0.104	——
Pseudo R^2^	0.112	——	0.112

Notes are the same as Table 3.

Entrepreneurship inherently involves risk-taking, and social trust can facilitate risk-sharing, promote information exchange, and build social capital, thereby enhancing entrepreneurial motivation. Moreover, trust plays a pivotal role in overcoming cognitive barriers and addressing institutional deficiencies. Consequently, social trust acts as a crucial intermediary in the relationship between corruption and entrepreneurship, aligning with Hypothesis 3.

In sum, the test results substantiate Hypothesis 3, confirming the mediating roles of both social networks and social trust in the effect of corruption on entrepreneurial decisions.

## 6. Conclusion and policy recommendations

While prior studies have highlighted the macro-level impact of corruption on reducing entrepreneurial activity, this study adopts a micro-level perspective to examine the nuances of this relationship. Our findings reveal a nuanced “inflection point” effect between the degree of corruption and resident entrepreneurship, but this effect is subtle due to the relatively low corruption inflection point. In general, higher levels of corruption significantly deter residents from pursuing entrepreneurial ventures. Furthermore, this inhibitory effect is particularly pronounced in “survival-oriented” entrepreneurship and among individuals operating outside of formal state systems, as opposed to “opportunity-oriented” entrepreneurship and those with ties to government or state assets. Our analysis also uncovers significant partial mediating effects of social networks and social trust on the corruption-entrepreneurship relationship.

Based on these findings, we propose the following policy recommendations: Firstly, strengthen anti-corruption efforts and institutional support. Relevant regulatory agencies should focus on enhancing the transparency and integrity of business registration, property rights protection, and tax oversight. Secondly, prioritize anti-corruption within government-related sectors. Public administration offices and supervisory bodies should address corruption within state-controlled institutions and enterprises. Thirdly, implement targeted support for survival-oriented entrepreneurs. Financial regulatory authorities and local business development agencies should work to reduce financial and bureaucratic barriers for smaller-scale, necessity-driven entrepreneurs. Fourthly, enhance regulations on public officials’ private social activities. Local civil affairs departments should oversee officials’ social engagements, promoting an environment where entrepreneurs are free from undue social burdens, allowing for more focused business interactions. Finally, bolster public trust and government credibility. Community outreach programs led by public information offices and educational institutions can promote social trust.

This study provides insights into how corruption affects resident entrepreneurship but has some limitations. On one hand, the use of 2015 CGSS data may not reflect recent institutional changes or anti-corruption efforts. Future research could utilize more recent or longitudinal data to capture these dynamics. On the other hand, while social networks and social trust are explored as mediators, other factors like financing access and regulatory costs are not considered. Including these in future studies could offer a more comprehensive view of corruption’s impact on entrepreneurship.
